# The Role of Transparency, Trust, and Social Influence on Uncertainty Reduction in Times of Pandemics: Empirical Study on the Adoption of COVID-19 Tracing Apps

**DOI:** 10.2196/25893

**Published:** 2021-02-08

**Authors:** Andreas Oldeweme, Julian Märtins, Daniel Westmattelmann, Gerhard Schewe

**Affiliations:** 1 University of Muenster Muenster Germany

**Keywords:** Uncertainty Reduction Theory, URT, COVID-19, tracing app, mobile health care adoption, DCA-transparency, social influence, initial trust, public health, eHealth, communication, trust, surveillance, monitoring, app, empirical, risk, use

## Abstract

**Background:**

Contact tracing apps are an essential component of an effective COVID-19 testing strategy to counteract the spread of the pandemic and thereby avoid overburdening the health care system. As the adoption rates in several regions are undesirable, governments must increase the acceptance of COVID-19 tracing apps in these times of uncertainty.

**Objective:**

Building on the Uncertainty Reduction Theory (URT), this study aims to investigate how uncertainty reduction measures foster the adoption of COVID-19 tracing apps and how their use affects the perception of different risks.

**Methods:**

Representative survey data were gathered at two measurement points (before and after the app’s release) and analyzed by performing covariance-based structural equation modeling (n=1003).

**Results:**

We found that uncertainty reduction measures in the form of the transparency dimensions disclosure and accuracy, as well as social influence and trust in government, foster the adoption process. The use of the COVID-19 tracing app in turn reduced the perceived privacy and performance risks but did not reduce social risks and health-related COVID-19 concerns.

**Conclusions:**

This study contributes to the mass adoption of health care technology and URT research by integrating interactive communication measures and transparency as a multidimensional concept to reduce different types of uncertainty over time. Furthermore, our results help to derive communication strategies to promote the mass adoption of COVID-19 tracing apps, thus detecting infection chains and allowing intelligent COVID-19 testing.

## Introduction

### Background

At the onset of the COVID-19 pandemic, people, organizations, and governments worldwide were plunged into uncertainty [1], leading to changes in everyday behavior [2,3]. To control the pandemic and protect lives, the authorities have implemented different policies, which range from recommendations (eg, enhanced hand and respiratory hygiene or ventilating rooms) over relatively mild measures (eg, maintaining social distance or mandatory face masks) to far-reaching interventions in civil rights (eg, restrictions in human mobility or lockdowns) [4-6].

Fighting the pandemic effectively is a complex challenge, since limited resources in the health care system and restrictions in everyday life need to be considered simultaneously [7,8]. Consequently, measures to control the pandemic have to be coordinated [9]. Especially when there is no widely available appropriate vaccine, testing, contact tracing, and isolation are considered to be the most essential measures against COVID-19 [9,10]. From a health care management perspective, testing is a key element and provides valuable information regarding the spread of the virus over time [9]. However, limited testing budgets and test resources such as trained personnel, indicator reagents, polymerase chain reaction devices, or laboratories are the bottlenecks that limit test capacities [11]. Eames and Keeling [12] already showed during the first severe acute respiratory syndrome–related coronavirus pandemic in 2003 that contact tracing, followed by treatment or isolation, is an effective measure in the fight against infectious diseases when testing capacities are limited [13]. Nevertheless, the effectiveness of contact tracing depends primarily on timely and comprehensive collection and processing of data [14,15]. Manual contact tracing only meets these requirements to a limited extent, as it is time and resource intensive, and prone to errors [16]. The resulting urgent need for action toward digital solutions has become evident, as health authorities are collapsing under the burden of manual contact tracing [16].

Contact tracing apps have attracted discussions among politicians, epidemiologists, and the public. These apps aim to systematically identify COVID-19 infection chains and allow a timely and targeted implementation of further measures such as testing and quarantine [14]. Simulation models indicate that digital contact tracing is more efficient compared to manual solutions and has the potential to prevent up to 80% of all transmissions [14,17]. To realize this potential, a majority of the population has to use the same or compatible COVID-19 tracing apps [18]. Achieving mass but voluntary acceptance of the technology is a substantial challenge for several governments [19]. The first positive effects are already expected at a penetration rate of about 20% [15]. However, the penetration rate in several countries as of October 2020 was far below this value, which illustrates the imperative for action to realize the potential of contact tracing apps [20]. Although previous studies have focused on the effectiveness [14,17,19,21] or technical specifications of COVID-19 tracing apps [22], none have examined the factors that affect a rapid and widespread adoption of a COVID-19 tracing app after its release.

Thereby, the use of COVID-19 tracing apps may be related to various uncertainties. These uncertainties can be classified into general health-related COVID-19 concerns and app-specific risks in the form of performance risks and privacy risks that arise because the apps require the processing of sensitive user data [22-24]. In addition, social risks can occur as people might fear social pressure or social exclusion from using or not using the tracing app [25]. According to the Uncertainty Reduction Theory (URT), these uncertainties can be reduced by appropriate means such as transparent communication, social influence, and trust [26-29]. Thereby, the uncertainty reduction means can foster the adoption process of technologies in general [28] and COVID-19 tracing apps in particular. Since COVID-19 tracing apps are mainly released by governments or in cooperation with governmental institutions, trust in the government was examined in addition to the initial trust in a COVID-19 tracing app [24]. For a deeper understanding of the factors at play, the following research questions (RQs) were examined:

- RQ1: How do transparency, social influence, trust in the government, and initial trust in a COVID-19 tracing app affect the adoption process of the app?
- RQ2: What effect does the actual use of COVID-19 tracing apps have on uncertainties in the form of perceived privacy, performance, and social risks, as well as general COVID-19 concerns?

To address the two RQs, we developed a theoretical model based on URT. For testing the model, a representative sample of potential users of a COVID-19 tracing app were surveyed at two different times (1 week before and 4 weeks after the launch of the app) via structured online surveys. Based on this data, we performed covariance-based structural equation modeling (CB-SEM; n=1003). In the following sections, we provide information about COVID-19 tracing apps, explain the theoretical foundation, and derive the hypotheses.

### Contact Tracing Apps as a Countermeasure Against COVID-19

This brief review aims to outline the characteristics of automated contact tracing apps for identifying contacts at risk and controlling disease transmission in humans. So far, several countries and some regions have developed and introduced independent COVID-19 tracing apps, which differ in administrative procedure and technical configuration [21]. Two major technical approaches exist: (1) GPS data is used to determine whether individuals, respective to their devices, were located within a geographical proximity for a defined period of time and (2) Bluetooth Low Energy is used to track the concrete proximity and exchange encrypted tokens with other devices in the defined proximity [30,31]. In both cases, data is used to notify people that have been in contact with a person who is infected. The recorded data is either stored on central servers (eg, the tracing app of France) or decentralized locations (eg, the tracing app of Germany) on the particular device [31]. Beside the technical configuration, tracing apps also differ in terms of administrative procedure. Although European tracing apps have been voluntarily used so far, some countries (eg, China) require citizens to install the app [24,32]. Moreover, the source code might be published or withheld by the developers (open source policy). Despite these options, regions need one specific COVID-19 tracing app or at least a suitable interface linking the different apps to achieve a sufficient adoption rate and alert people who are possibly infected [22,33]. A frequently updated overview of the COVID-19 tracing apps used in different regions and their characteristics is provided by MIT Technology Review [20]. Although Trang et al [22] showed that app design influences the likelihood of mass acceptance, there is a lack of evidence to what extent administrative aspects affect the (mass) adoption of COVID-19 tracing apps.

### Uncertainty Reduction Theory

URT [26] originally addressed the initial interactions between strangers from a communication science perspective. The core assumption states that individuals face uncertainties in interactions with unknown partners, and individuals attempt to reduce these uncertainties. Berger and Calabrese [26] described uncertainty as a state in which a person is confronted with several alternatives concerning a stranger’s behavior. More alternatives make the individuals feel more uncomfortable because the other person’s behavior is harder to predict [34]. Although URT was initially developed to explain initial interactions between individuals, the theory has been applied to other contexts such as recruiting processes [35], computer-mediated communication [36], online commerce [37], or organizational behavior [38]. Hence, URT is not only limited to the interaction of individuals but is also useful in other settings. For instance, Venkatesh et al [28] demonstrated that URT is suitable for explaining the technology-supported communication of individuals and institutions in an e-governance setting. Beyond, URT is also suitable in crisis situations in general and in the current COVID-19 pandemic in particular [29].

The application of URT is appropriate in times of COVID-19 since the situation is marked by various far-reaching uncertainties. Looking at COVID-19 tracing apps, different uncertainties are apparent. First, health care technologies in general often bear uncertainties concerning data privacy [39]. These uncertainties are also identified in cases of COVID-19 tracing apps, as they require the processing of sensitive personal data [22,30]. Individuals fear that their privacy will be violated and cause undesirable outcomes such as governmental surveillance [30,40]. Moreover, people are concerned that personal data is used to impose quarantine or restrict access to public places for people who do not use a COVID-19 tracing app [24]. Second, uncertainties about the true performance and functionality of tracing apps are apparent [23]. Using a mobile app to contain a pandemic is new to individuals in most countries. Hence, they cannot draw on past experiences and might question its utility (eg, false alerts or only few people using the app). Third, social risks are recognizable as people might fear social pressure or social exclusion from using or not using the tracing app [25]. Beside tracing app–related uncertainties, general health-related COVID-19 concerns arise from the pandemic itself. The main aspects of the four uncertainties considered are summarized in Table 1. In addition, the four described uncertainties are further reinforced by unverified information and fake news [41-43].

**Table 1:** Summary of relevant uncertainties in the context of COVID-19 tracing apps.

Relevant uncertainties	Related to tracing apps	Description
Privacy risks	Yes	Individuals are uncertain about data security (ie, possible data leaks or misuse by third parties). Hence, tracing apps are perceived as risky because they bear the potential loss of control over personal data [22,44].
Performance risks	Yes	Individuals are concerned that the product may not work and perform as it was designed and advertised. As a result, people are uncertain whether enough people will use apps for contact tracing and whether the technology will work as expected [23].
Social risks	Yes	Individuals might fear potential loss of status in one’s social group for using or not using the app. In addition, forced quarantine might lead to social isolation [25].
COVID-19 concerns	No	Individuals worry about negative impacts arising from the COVID-19 pandemic. Fear and anxiety about a new disease, for their own health and their relatives, can be overwhelming [45].

### Uncertainty Reduction Measures

According to URT, individuals reduce uncertainties by passive (observing), active (target-orientated search), and interactive (interaction with the stranger) information-seeking approaches [26,27]. We discuss transparency, social influence, and (initial) trust, as these factors facilitate individual’s information-seeking strategies [28,46-48].

Notably for passive and active strategies, individuals rely on accessible and valuable information [46]. To enable people to reduce uncertainty through observation or targeted research, information must be available. If no information is obtainable, people cannot reduce uncertainties through observation or targeted research. Therefore, transparency is examined as an enabler for passive and active information-seeking strategies. We defined transparency as “the perceived quality of intentionally shared information from a sender” [49]. Drawing on recent transparency research, transparency is best understood as a multidimensional construct consisting of disclosure, clarity, and accuracy of information [49-51]. In the context of this study, disclosure is the perception that sufficient relevant information about a COVID-19 tracing app is timely and accessible. Similarly, clarity is the perception that the received information about a COVID-19 tracing app is comprehensible and lucid. For instance, the disclosure of a huge amount of information cannot be considered transparent if the information is not understandable for individuals (eg, because the information is cryptic and only consists of the technical code of the COVID-19 tracing app). This information would hinder an individual’s ability to effectively perform active and passive information seeking. Lastly, accuracy is the perception that the information about a COVID-19 tracing app is correct [49]. The apparent incorrectness of information would not lower uncertainty but might lead to concerns about hidden governmental intentions. Notably in the context of a pandemic, each transparency dimension contributes to the reduction of uncertainty, as individuals rely on sufficient, relevant, timely, clear, and accurate information to observe the unknown technology and to actively search for information [29].

Furthermore, interactive information-seeking approaches have been shown to be more efficient than passive or active strategies in reducing uncertainty [46]. As it is not possible to interact with COVID-19 tracing apps before they are released or to directly communicate with the people responsible for the app, people seek alternatives for interactive information gathering. Therefore, individuals may communicate with their peers who are also affected by the decision whether to use the app or not. This, in turn, has to be regarded as another active information-seeking approach rather than an interactional strategy. Although communication with the social environment is interactive, the social environment is not the publisher of the app, and therefore, referring to URT, social influence is an active information-seeking approach. Social influence is expected to reduce people’s uncertainty about COVID-19 tracing apps, and it is defined “as the degree to which an individual perceives that important others believe he or she should use the new system” [52]. By knowing the preferences of their social environment, individuals’ attitudes toward using the app might be affected.

Lastly, trust is shown to reduce uncertainties and risks in different settings [53,54], and it is defined as “a psychological state comprising the intention to accept vulnerability based upon positive expectations of the intentions or behaviors of another” [55]. We distinguish between initial trust in COVID-19 tracing apps and individuals’ trust in their government. Several positive links to uncertainty reduction exist for initial trust in new technologies. For example, in e-commerce, trust lowers customers’ uncertainties about vendor behavior [56,57]. Additionally, initial trust reduces citizens’ uncertainties in the wake of e-governance [25]. However, initial trust might change in the actual use of the app and become strengthened or weakened according to the specific experiences encountered [58]. In addition to initial trust, individuals’ trust in their government is another means to reduce uncertainties. As most COVID-19 tracing apps are published by governments, trust in the administration might reduce fears related to app use [59]. People’s trust in the government is expected to be relatively stable and not fundamentally changeable in the short term [60,61].

### Transparency and Initial Trust

Based on the transparency and trust literature, it is widely believed that transparency perceptions are positively related to trust [62]. This is shown by Schnackenberg et al [63] who explored the positive role of employees’ transparency perceptions (disclosure, clarity, accuracy) in the context of employees’ trust in their manager in organizational settings. Rawlins [51] also showed a positive link between transparency and employee trust, and highlighted the mutual relation between transparency and trust. Regarding the consequences of corporate scandals, transparency can be used as a strategic tool to restore stakeholder trust in firms [64]. In financial markets, transparency is shown to influence citizens’ trust in central banks [65]. In the case of COVID-19 tracing apps, a certain degree of transparency must be achieved for people to trust the app and use the technology [24]. The formation of peoples’ initial trust in COVID-19 tracing apps relies on the quality of available information as long as there are no prior interactions between citizens and the app [53,57]. Fulfilling certain information needs (eg, by providing sufficient clear and accurate information) enables people to initially trust a COVID-19 tracing app.

- Hypothesis (H)1: (a) Disclosure, (b) clarity, and (c) accuracy are positively related to individuals’ initial trust in a COVID-19 tracing app.

### Trust in the Government and Initial Trust

Trust transfer theory states that individuals’ trust in a specific area can influence initial trust in other domains that are believed to have certain links to the known and trusted domain [66]. For instance, Lu et al [67] demonstrated that customers’ trust in internet payment in general influences trust in mobile payment services. As the majority of COVID-19 tracing apps are published by government institutions, trust in the government might affect initial trust in a COVID-19 tracing app. Peoples’ trust in the government is defined as the “perceptions regarding the integrity and ability of the agency providing [a] service” [53]. When people believe that the government is generally acting in citizens’ best interest and when citizens perceive the government agencies as capable to appropriately offer services, the initial trust in a COVID-19 tracing app is strengthened [53]. Recent studies on COVID-19 tracing apps noted that trust in the government influences peoples’ attitude toward the specific app [59,68]. In addition, a main reason for general negative attitudes against COVID-19 tracing apps is a lack of trust in the government [68]. Therefore, based on trust transfer theory, trust in the government fosters peoples’ initial trust in a COVID-19 tracing app [28].

- H2: Trust in the government is positively related to peoples’ initial trust in a COVID-19 tracing app.

### Social Influence, Initial Trust, and Intention to Use

As previously stated, social influence can also aid the understanding of uncertainty reduction, as it might function as a substitute for interaction with the unknown and not yet available technology. Therefore, social interaction serves as an active means to gather information. The presumed reactions of the social environment will influence an individual’s attitude and behavior in a technology adoption context [52]. In terms of URT, people access their social environment as an active information-seeking means by interacting with their peers to exclude possible consequences of using or not using the specific technology. In this sense, social interaction, just like transparency, is a means of obtaining information and excluding alternatives and, hence, serves to reduce uncertainties. Li et al [69] showed that social influence is an important factor for the formation of initial trust and is therefore contributing to the exclusion of expectable negative outcomes such as perceived risks. Against this background, we argue that initial trust in a COVID-19 tracing app is not only influenced by transparency and trust in government, but is also affected by social influence.

- H3a: Social influence is positively related to individuals’ initial trust in a COVID-19 tracing app.

In addition, it is well known that social influence is an important antecedent of intention to use new technologies [70-72]. Being part of social groups (eg, family or colleagues) creates pressure on individual behavior, as people try to behave in accordance to established standards [73]. In health care settings, it has been shown that social influence is, for example, leading to smoking cessation [74] or supporting to maintain a diet [73,75]. Besides positive effects, the peer group might also foster negative behaviors such as drug abuse [76]. Therefore, social influence is a major factor to consider in the adoption process of health care technologies. This is particularly reinforced in the case of preventive behaviors like using tracing apps whose positive effect is not directly evident [73]. Therefore, we expect that social influence is not only influencing one’s initial trust in COVID-19 tracing apps but also impacts one’s intention to use the technology.

- H3b: Social influence is positively related to individuals’ intention to use a COVID-19 tracing app.

### Initial Trust and Intention to Use

In the technology acceptance literature, trust has been shown to be positively correlated with the intention to use technology [69]. For instance, Nicolaou and McKnight [77] demonstrated that individuals’ trusting beliefs increase the intention to engage in interorganizational information exchange. Furthermore, (initial) trust is identified to be an antecedent of citizens’ intention to use e-governance services [28,53]. Parker et al [24] argued that the successful launch of mobile apps to fight the COVID-19 pandemic in democratic countries relies on the ability to ensure peoples’ trust in the technology. Based on URT, we argue that initial trust is a means to exclude potential negative behavior of the technology provider. Citizens who trust a COVID-19 tracing app estimate the probability of deceitful intentions as low.

- H4: Initial trust in a COVID-19 tracing app is positively related to individuals’ intention to use it.

### Intention to Use and Actual Use

According to the theory of planned behavior [78], established technology acceptance theories (eg, technology acceptance model [TAM] or unified theory of acceptance and use of technology [UTAUT]) [52,79], and the application of URT in the technology context [28], explains that individuals’ actual use of new technology is influenced by individuals’ intention to use the technology [80]. This relationship is also expected to be applicable in the context of COVID-19 tracing apps.

- H5: Intention to use is positively related to individuals’ actual use of a COVID-19 tracing app.

### Actual Use and Uncertainty Reduction

Referring to URT, uncertainties concerning data privacy, app performance, social consequences, and general COVID-19 concerns are decreased by the aforementioned means during the adoption process. The actual use of a COVID-19 tracing app is the only available interactive information-seeking possibility for individuals. Therefore, it is effective as it involves a direct interaction with the unknown technology [27]. This interaction enables individuals to discard uncertainties such as performance uncertainties (eg, functionality and handling) of COVID-19 tracing apps [29]. However this mean can obviously only be used after the app has been released. The investigation of the relationship between actual use and the reduction of different forms of uncertainty further addresses the applicability of URT for technology acceptance in uncertain environments.

- H6: The actual use of a COVID-19 tracing app is positively related to the reduction of (a) privacy risks, (b) performance risks, (c) social risks, and (d) COVID-19 concerns.

**Figure 1:**
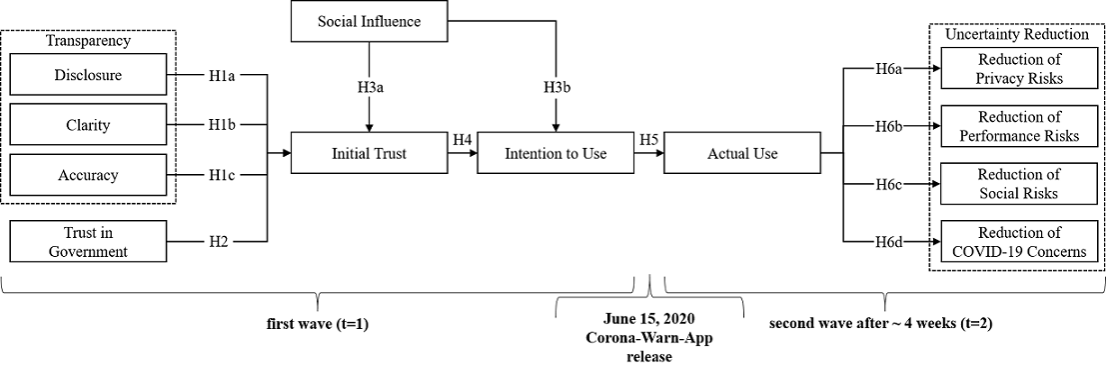
Proposed research model. H: hypothesis.

The proposed research model is summarized in Figure 1.

## Methods

### Data and Procedure

To investigate the adoption process of COVID-19 tracing apps and test the theoretical model, data on the German “Corona-Warn-App” were collected via the panel platform respondi in two waves using structured online surveys. Members of this panel voluntarily agree to receive invitations to scientific surveys and may unsubscribe or delete their personal information at any time. Participants were assigned a randomly generated identifier, allowing us to match the results of both surveys. Nevertheless, since a third party (respondi) collected the data for both waves, we did not have direct contact with participants or access to identifying participant information. In addition, the survey did not collect any personal identifying information. Consequently, we were able to guarantee the anonymity and privacy of the participants at all times and acted in accordance with the ethical principles of the German Research Foundation.

The data collection of the first wave (t1) lasted 1 week and was completed 1 day before the app was released on June 15, 2020. Prior to the survey, the participants received the official information from the Federal German Government about the app [81]. For detailed information, see Multimedia Appendix 1 [81]. The app was developed on Android and iOS platforms by Robert Koch-Institut in conjunction with the private companies SAP, Deutsche Telekom, and other partners on behalf of the German government [81]. For the administrative conditions, the source code is publicly available on GitHub [82,83]. Furthermore, no registration is required to run the app, and the use is voluntary. The app uses Bluetooth Low Energy technology to record contact between people. Considering data privacy and security concerns, the devices exchange temporary encrypted random codes (Bluetooth ID) with each other [84]. The use of random codes prevents conclusions from being drawn about individual users or their specific locations. These codes are stored decentralized on the mobile devices, and the tracing data collected are automatically deleted after 14 days [85].

Subsequently, the participants received a quantitative questionnaire concerning their evaluations and perceptions of the app as well as demographical information. In the second survey wave (t2), the same participants were surveyed again 4 weeks after the app was released. The actual use of the app was queried in addition to their evaluations and perceptions.

To maintain data quality and ensure scale validity, we included three attention checks and screened out participants that failed these tests. Finally, 1373 individuals completed the first survey, and 1050 participated in the second wave, yielding a completion rate of 76.47% (1050/1373). Owing to four “knock-out” criteria regarding low response times, missing data, suspicious response patterns, and outliers, 47 participants were excluded from the analysis to ensure data quality [86]. A final sample size of 1003 was then obtained. This sample is representative of Germany in terms of gender, age, and education (see Table 2).

**Table 2:** Representativeness description.

Characteristic		Germany, %	Sample, n (%)
**Gender**			
	Male	50	490 (48.85)
	Female	50	512 (51.05)
	Divers	0	1 (0.10)
**Age (years)**			
	14-29	22	190 (18.94)
	30-39	16	138 (13.76)
	40-49	16	168 (16.75)
	50-59	20	214 (21.34)
	≥60	25	291 (29.01)
**Education**			
	Low	36	368 (36.69)
	Middle	31	315 (31.41)
	High	33	320 (31.90)
Adoption rate of Corona-Warn-App (August 2020)^a^		27	379 (37.80)

^a^Calculation of adoption rate in Germany: 16.6 million downloads / approximately 62 million smartphone users (source: Statistisches Bundesamt [87,88], and Robert Koch-Institut [83]).

### Measures

To test the proposed research model, we used established scales that have been validated in previous studies. Except for demographics, use behavior (binary), and control variables (gender, age, education), the participants rated all items on 5-point Likert scales. Intention to use was measured by a 3-item scale [52]. For initial trust in the Corona-Warn-App, we built on a 5-item scale developed by Koufaris and Hampton-Sosa [58]. Trust in government was examined through a 4-item scale adapted from Bélanger and Carter [53]. Transparency (reflecting individuals’ perceptions of information quality) was adopted from Schnackenberg et al [63], and each dimension was based on 4 items. Privacy risks was measured on a 5-item scale developed by Rauschnabel et al [44], and a 5-item measure was adopted from Featherman and Pavlou [89] to assess performance and social risks. Finally, general COVID-19 concerns were measured through a 6-item scale by Conway et al [45].

We calculated differences for the four dimensions of risk perceptions between the two survey waves to measure the change in the perceived risk assessments. The differences were calculated using the following formula: difference variables = risk perception_(t1)_ – risk perception_(t2)_. The means, SDs, and correlations for all constructs are reported in Table 3. Age, gender, and education were used as controls.

**Table 3:** Mean, SD, and correlations.

Variables		Mean (SD)	1	2	3	4	5	6	7	8	9	10	11
**1. Disclosure**		3.158 (0.909)											
	Correlation		—^a^										
	*P* value		—										
**2. Clarity**		3.647 (0.834)											
	Correlation		0.645	—									
	*P* value		<.001	—									
**3. Accuracy**		3.566 (0.898)											
	Correlation		0.587	0.705	—								
	*P* value		<.001	<.001	—								
**4. Social influence**		2.841 (1.12)											
	Correlation		0.440	0.427	0.585	—							
	*P* value		<.001	<.001	<.001	—							
**5. Trust in government**		3.13 (0.979)											
	Correlation		0.332	0.334	0.505	0.454	—						
	*P* value		<.001	<.001	<.001	<.001	—						
**6. Initial trust**		3.147 (1.081)											
	Correlation		0.551	0.545	0.742	0.71	0.59	—					
	*P* value		<.001	<.001	<.001	<.001	<.001	—					
**7. Intention to use**		3.022 (1.444)											
	Correlation		0.434	0.429	0.619	0.685	0.466	0.803	—				
	*P* value		<.001	<.001	<.001	<.001	<.001	<.001	—				
**8. Actual use**		1.378 (0.485)											
	Correlation		0.288	0.307	0.377	0.419	0.355	0.512	0.595	—			
	*P* value		<.001	<.001	<.001	<.001	<.001	<.001	<.001	—			
**9. Privacy risk**		—											
	Correlation		–0.074	0.037	–0.033	–0.100	–0.047	–0.106	–0.070	0.224	—		
	*P* value		.02	.24	.30	.002	.14	<.001	.03	<.001	—		
**10. Performance risk**		—											
	Correlation		–0.063	–0.001	–0.055	–0.148	–0.008	–0.109	–0.091	0.169	0.595	—	
	*P* value		.045	.97	.08	<.001	.80	<.001	.004	<.001	<.001	—	
**11. Social risk**		—											
	Correlation		0.031	–0.044	–0.020	0.064	–0.023	–0.001	0.025	0.008	0.007	0.086	—
	*P* value		.32	.16	.54	.04	.47	.98	.43	.81	.83	.006	—
**12. COVID-19 concerns**		—											
	Correlation		0.070	0.081	0.038	0.057	0.042	0.04	0.033	0.011	–0.023	0.027	0.041
	*P* value		.03	.01	.23	.07	.19	.21	.30	.72	.48	.39	.19

^a^Not applicable.

Before conducting the structural equation modeling (SEM) analysis, we tested the reliability and validity of the measurement model. One item displayed poor factor loadings and was dropped (TR_5). All other factor loadings exceeded the threshold of 0.6. Internal consistency and composite reliability were assumed, as the Cronbach alpha met the quality criteria of >.7, and the average variance extracted exceeded 0.5 [90,91]. Composite reliability of all items exceeded the cut-off value of 0.6 [92]. The final questionnaire with all constructs, related survey items, their sources, and the aforementioned indexes is presented in Multimedia Appendix 2 [44,45,52,53,58,63,89,93].

### Data Analysis

We used the R-based JASP software (University of Amsterdam) environment to evaluate our proposed research model [94] and the lavaan code to conduct CB-SEM [95] analysis. Before performing the SEM analyses, we tested the fit, reliability, and validity of the applied model. The comparative fit index (>0.95), Tucker-Lewis index (>0.95), root mean square error of approximation (<0.08), and standardized root mean square residuals (<0.08) complied with the conventional cut-off criteria [96,97]. Based on Kline [98], the *χ*² / df ratios indicated a sufficient model fit across models (<3). Common method bias was not a problem, as the Harman single factor test indicated that only a variance of 27.6% were explained by a single factor consisting of all model items [99]. In summary, all fit indexes revealed a very good overall model fit (see Table 4), with all indicators reaching their respective thresholds.

**Table 4:** Covariance-based structural equation modelling results.

Items		β (SE)^a^	*P* value	Assessment of hypotheses	Index values	
**Hypotheses**						N/A^b^
	H^c^1a	.140 (.030)	<.001	Supported		
	H1b	–.028 (.041)	.45	Rejected		
	H1c	.375 (.035)	<.001	Supported		
	H2	.201 (.022)	<.001	Supported		
	H3a	.377 (.025)	<.001	Supported		
	H3b	.207 (.033)	<.001	Supported		
	H4	.670 (.035)	<.001	Supported		
	H5	.599 (.013)	<.001	Supported		
	H6a	.222 (.048)	<.001	Supported		
	H6b	.169 (.049)	<.001	Supported		
	H6c	.005 (.064)	.88	Rejected		
	H6d	.012 (.031)	.72	Rejected		
**Controls**				N/A	N/A	
	Age → PRPP^d^	–.090 (.002)	.005			
	Age → PR^e^	–.062 (.002)	.06			
	Age → SR^f^	–.004 (.002)	.91			
	Age → CC^g^	–.064 (.000)	.06			
	Gender → PRPP	–.001 (.047)	.96			
	Gender → PR	.000 (.048)	.99			
	Gender → SR	.015 (.062)	.63			
	Gender → CC	–.017 (.030)	.59			
	Education → PRPP	–.006 (.020)	.85			
	Education → PR	.000 (.021)	.99			
	Education → SR	.020 (.027)	.54			
	Education → CC	–.007 (.013)	.84			
**Indexes**		N/A	N/A	N/A		
	Comparative fit index				0.975	
	Tucker-Lewis index				0.972	
	RSMEA^h^				0.040	
	SRMR^i^				0.057	
	Chi-square (*df*)				1270.187 (491)	
	Chi-square / *df*				2.587	

^a^Standardized path coefficients; standard error of the estimators in parentheses.

^b^N/A: not applicable.

^c^H: hypothesis.

^d^PRPP: reduction of privacy risks.

^e^PR: reduction on performance risks.

^f^SR: reduction of social risks.

^g^CC: reduction of COVID-19 concerns.

^h^RSMEA: root mean square error of approximation.

^i^SRMR: standardized root mean square residuals.

## Results

**Figure 2:**
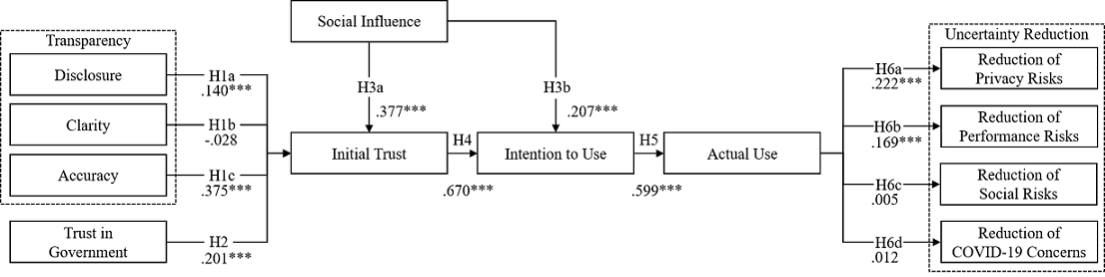
Results of structural equation model. H: hypothesis. ***P<.001.

The standardized path coefficients, significance levels, and fit indexes are summarized in Table 4. As illustrated in Figure 2, information disclosure and accuracy are positively related to initial trust, supporting H1a (β=.140; *P*<.001) and H1c (β=.375; *P*<.001). In contrast, H1b was rejected (β=–.028; *P*=.45), as information clarity shows no relation to initial trust. H2 was supported (β=.201; *P*<.001) as trust in governance and initial trust were positively related. Furthermore, there was support for H3a (β=.377; *P*<.001) and H3b (β=.207; *P*<.001), as the results showed a positive relation between social influence toward initial trust and intention to use. The observed relationship between initial trust and intention to use was positive, supporting H4 (β=.670; *P*<.001). We also found support for H5 (β=.599; *P*<.001), as intention to use was positively related to the actual use of a COVID-19 tracing app. Finally, we found a positive relationship between actual use and privacy and performance risks, thus supporting H6a (β=.222; *P*<.001) and H6b (β=.169; *P*<.001). In contrast, H6c (β=.005; *P*=.88) and H6d (β=.012; *P*=.72) were rejected as actual use was not related to social risks or COVID-19 concerns, respectively. The control variables gender and education were not related to the reduction of the four dimensions of uncertainty reduction, while age was negatively related to privacy risk reduction.

In addition to the hypothesized direct relationships between social influence and the intention to use the COVID-19 tracing app, we conducted a post hoc analysis to investigate the potential indirect effects of social influence on the intention to use the COVID-19 tracing app mediated by initial trust. The mediation effects were examined with the help of the procedure according to Baron and Kenny [100] and are depicted in Table 5. We found evidence that the indirect effect was significant (β=.253; *P*<.001). As the direct effect of social influence toward the intention to use has been shown to be significant before (H5), we postulated that the relationship of social influence and intention to use is partially mediated by initial trust.

**Table 5:** Mediating effect.

Effect	β (SE)^a^	*P* value	Mediation
Indirect effect: social influence → initial trust → intention to use	.253 (.022)	<.001	partial mediation
Total effect: social influence → intention to use	.460 (.029)	<.001	–

^a^Standardized path coefficients; standard error of the estimators in parentheses.

## Discussion

### General Discussion

In this study, we investigated how uncertainty reduction measures can foster the adoption of COVID-19 tracing apps and, consequently, the reduction of uncertainty perception. In this section, we discuss the antecedents of initial trust, intention, and actual use of the app, as well as the reduction of specific uncertainties. Based on URT, transparency and social influence are antecedents of initial trust. In terms of transparency, we found that initial trust in COVID-19 tracing apps is positively influenced by the disclosure and accuracy of information. However, accuracy had a considerably higher effect on initial trust than the disclosure dimension. This shows that, although it is important to receive sufficient information, the perceived validity of the information is crucial. Unexpectedly, we found no effect between information clarity and initial trust in the Corona-Warn-App. This may be due to the peculiarities of the COVID-19 pandemic, as people likely became used to constantly encountering new complex information and thus accepted a lower level of information lucidity. Despite the missing effect between clarity and initial trust, our findings are consistent with the existing transparency-trust literature [51,62,63].

As proposed, social influence positively affects individuals’ initial trust. The integration of social influence in the URT context reveals that social influence serves as an active information-seeking strategy, thus meeting the demand of Venkatesh et al [28] to integrate UTAUT variables into URT. Especially in situations where direct interaction with the unknown technology is not possible, the communication with peers becomes important. In addition, we identified a positive relation between social influence and intention to use. This is in line with technology acceptance and health care literature [52,70,73]. In addition, we were able to show that initial trust partially mediates the relationship between social influence and intention to use COVID-19 tracing apps. Furthermore, we examined the effect of trust in the government on initial trust in COVID-19 tracing apps and found a positive relationship between these concepts. This is consistent with the trust transfer theory and current studies on COVID-19 tracing apps [59,68]. It is important to note that trust in government has a smaller effect on individuals’ initial trust compared to transparency and social influence. Therefore, people who are critical of the government can still develop initial trust in the app through other short-term influenceable means such as transparent communication.

Additionally, we observed a positive relation between initial trust and intention to use. This result is consistent with URT [27,28] and confirms the common understanding of trust in the context of technology acceptance (for a meta-analysis, see Wu et al [101]). As expected, people who have a high intention to use a COVID-19 tracing app are more likely to use it. Nevertheless, our results also revealed, as most studies have, that an intention-behavior gap exists [102].

Considering uncertainty reduction specifically, we found that the actual app use increases COVID-19 tracing app–related uncertainty reduction. Individuals’ uncertainty reduction of perceived privacy and performance risks was significantly increased by using the app. Thus, we found support for Trang et al [22], who stated that data privacy and app performance (benefits) need to be considered in the development of tracing apps. In addition, our results did not indicate a reduction of social risks nor a reduction of general COVID-19 concerns. As COVID-19 concerns span broader health-related fears, they cannot be solely linked to the functionality of the app or interaction with it. Tracing apps do not provide direct protection but are mainly intended to identify infection chains to implement further appropriate actions such as intelligent testing and quarantine [9]. This explains why the use of a COVID-19 tracing app has no impact on the reduction of these general health-related fears. Further, it indicates that people using tracing apps are not getting more reckless but still recognize the virus’s threat. Regarding social risks, the use and nonuse of the app is less visible to nearby people than wearing a face mask or complying with social distance regulations. Therefore, individuals’ actual use behaviors might be unrelated to social consequences as long as the use of such an app is not mandatory, for example, to use public transportation or enter restaurants or other places. For the controls, we found that age was negatively correlated to the reduction of privacy risks. This effect is rather small and in line with research emphasizing that privacy concerns are more pronounced and stable among older people than among younger individuals [103].

### Theoretical Implications

Our study design and findings contribute to the literature in several ways. First, we demonstrated with our study design how mass adoption problems can be investigated over time in the health care management context using the example of a COVID-19 tracing app. By applying URT, we contributed to its empirical validation in general and introduced it to the field of health care management. The application is particularly valuable in the health care context, as this area is characterized by uncertainties that may lead to serious and far-reaching consequences, as is apparent in times of the COVID-19 pandemic [104]. Second, it was shown that interactive information-seeking strategies, such as app use, are appropriate for reducing related uncertainties (eg, privacy and performance risks). By collecting the data in two measurement periods (before and after the release of the app) and calculating difference variables to quantify the uncertainty reduction, we validated the impact of the use of a technology on uncertainty reduction. The use of specific uncertainty reductions as outcome variables is theoretically stronger for URT than the use of outcome variables such as satisfaction proposed by Venkatesh et al [28]. Third, further theoretical contributions were made by integrating recent transparency research [49] into URT. Thereby, our results highlighted the importance of considering transparency as a multidimensional construct [49]. Transparency perceptions are essential as they form the basis for active and passive information-seeking strategies. By using the recent DCA-transparency scale [63], we further elaborated on the role of transparency (ie, information quality) in URT as proposed by Venkatesh et al [28]. Finally, it was shown that trust transfer theory holds true in the investigated setting. Although trust in the government is not a major antecedent for initial trust in COVID-19 tracing apps, individuals’ trust in the government should still be considered in governmental technology publishing.

### Implications for Practice

The adoption rates of voluntary COVID-19 tracing apps differ largely among countries and are mostly below the critical thresholds, which hinders their effectiveness [14,20]. To improve acceptance, governments can adopt the following implications in their communication strategies. First, governments that introduce a voluntary COVID-19 tracing app (or other technologies) should engage in a transparent communication process. A supply of sufficient information, which must be perceived as accurate, is thus required. However, transparent communication only works if the service itself exceeds certain standards such as data privacy and security [22]. Second, interactive information-seeking strategies of individuals must be managed. These strategies (eg, app use) are shown to be efficient in terms of uncertainty reduction. Hence, governments should provide appropriate formats to enable interactive information seeking before release. Such formats can be demo versions, realistic previews, question and answer sessions, or even hackathons. Finally, our findings are extendable to other technologies and settings. For example, if there are other digital trends in the health care system (eg, digital health record or video doctor), our results can be applied to achieve (voluntary) technology (mass) acceptance. Whenever governments or organizations develop and publish new services (eg, disaster alarm app), other uncertainties such as financial risk, time risk, or psychological risk may arise and should be considered. The conscious management of the (transparent) publication process can promote a successful launch of a technology. By understanding the multidimensional nature of perceived information quality, both organizations and governments can reflect and develop their own technology implementation strategy. Hence, many of the implications outlined here may also be relevant to future pandemics and public health crises.

### Limitations and Future Research

Although the results of this study provide important insights, the study has some limitations. As the results are based on data related to the German COVID-19 tracing app, the generalizability of our findings for other regions may be restrained due to cultural differences. Thus, future research should expand this study by including other countries. Further, actual app use was self-reported by the participants and might be untrue in some cases. However, the app adoption rate in our sample was comparable with the adoption rate in the German population during the second survey wave (see Table 2). To advance URT, researchers can examine the communication channels that are most suitable to ensure transparency and reduce different uncertainties. After some studies have dealt with the design [105], the technical configuration [22], and the ethical guidelines [106], we studied the requirements for adequate app implementation and communication. Therefore, future research should investigate means to ensure mid- and long-term app acceptance and use.

For most of the population, the Corona-Warn-App was a new concept at the time of its release. Since then, the app and its functionality have become relatively well known and widespread. For this reason, follow-up research should investigate the role of descriptive norms (ie, how others actually behave) besides subjective norms, which we have investigated in the form of social influence (ie, how important others think one should behave), for the adoption process [107].

Moreover, the data underlying this study originated a few days (t1) before and 4 weeks (t2) after the launch of the COVID-19 tracing app in Germany and, thus, between the first and second waves of infection. In the meantime, various measures against the pandemic have been implemented, and more information about the virus, its spread, and mortality are available. These insights should be considered in follow-up studies. For example, the distribution and adoption of new SARS-CoV-2 vaccines represent a milestone in the fight against the pandemic. Therefore, follow-up studies should examine whether these insights influence the use of the COVID-19 tracing app and uncertainty perceptions.

### Conclusion

A key strategy in fighting the COVID-19 pandemic is the testing and subsequent isolation of individuals who are potentially infected. The automatic contact tracing via mobile apps offers an important contribution to the decision of which people need to be tested with regard to limited testing capacities. Our study offers original insights on the factors driving the mass acceptance of COVID-19 tracing apps to identify infection chains and control the pandemic. Building on URT and through a longitudinal empirical study on the adoption process, we investigated how uncertainty reduction measures affect the adoption of COVID-19 tracing apps and how their use affects the perception of different risks. We analyzed representative data through CB-SEM. The results revealed that the transparency dimensions of disclosure and accuracy, as well as social influence, trust in government, and initial trust positively affect the adaptation process, whereas no effect was observed for the transparency dimension clarity. Further, we showed that the actual use of COVID-19 tracing apps reduces the perceived uncertainty regarding performance and privacy risks, but no effect on the reduction of social risks and COVID-19 concerns was identified. Finally, we derived theoretical and practical implications concerning the communication strategy of contact tracing apps in particular and for health care technologies in general.

## Appendix

Multimedia Appendix 1 Background information for participants.

Multimedia Appendix 2 Items of the survey and their sources.

## Abbreviations

CB-SEM: covariance-based structural equation modeling
H: hypothesis
RQ: research question
SEM: structural equation modeling
TAM: technology acceptance model
URT: Uncertainty Reduction Theory
UTAUT: unified theory of acceptance and use of technology
