# Supplementary material for: The Role of Transparency, Trust, and Social Influence on Uncertainty Reduction in Times of Pandemics: Empirical Study on the Adoption of COVID-19 Tracing Apps
Source: J Med Internet Res. 2021 Feb 8;23(2):e25893. doi: 10.2196/25893 (PMC7872328; doi:10.2196/25893)
Supplement: Multimedia Appendix 1 [file jmir_v23i2e25893_app1.docx]

Background information for participants.

Please read the following information carefully

Why do we need a Corona-Warn-App?

The app should help to curb the spread of COVID-19. It documents the digital encounter between two smartphones. This allows the app to quickly inform you if you had contact with a person diagnosed with COVID-19. The faster you receive this information, the lower the risk that many people will become infected. That is why the app is an effective means of curbing the corona virus, along with hygiene measures such as hand washing, social distancing and using everyday face masks. The Federal Government supports the app because it serves the protection and health of the community.

How does the Corona-Warn-App work?

The Corona-Warn-App uses Bluetooth technology to measure the distance and duration of the encounter between people who have installed the app. The smartphones "remember" encounters if the criteria determined by the RKI on distance and time are met. The devices then exchange temporary encrypted random IDs. If people using the app test positive for the corona virus, they can inform other users on a voluntary basis. Then the random IDs of the person diagnosed with COVID-19 are made available to all people who are using the Corona-Warn-App. If you have installed the app, it will check whether you have had contact with the person diagnosed with COVID-19 for you. This check is only performed on your smartphone. If it is positive, the app will display a warning. At no point in time does this procedure allow connections to be made to you or your location.

How secure is such an app?

In developing the Corona warning app, the German government is pursuing an approach that is based on voluntariness, complies with data protection requirements and ensures a high level of IT security. In the event of infection, the corresponding message is sent anonymously to the contact person.

Who is behind the Corona-Warn-App?

The Corona-Warn-App is a project commissioned by the Federal Government. The companies Deutsche Telekom and SAP have developed the application, based on a distributed software architecture. The Fraunhofer-Gesellschaft and the Helmholtz Center for Information Security (CISPA) provided advice and support. Another important aspect of the application is interoperability. This means that other European tracing apps should also be compatible with the German application. The two companies Google and Apple provide Bluetooth interfaces, so-called APIs. This programming interface offers other programs the possibility to connect to the system. In order to meet the requirements for data protection and data security, the Federal Office for Information Security (BSI) and the Federal Commissioner for Data Protection and Freedom of Information (BfDI) were also involved. The Robert Koch Institute plays a dual role in the Corona-Warn-App: it provides specialist expertise for the development of the app, and as publisher is also responsible for carefully checking the requirements for data protection and data security.

Note. Information and text retrieved from the Robert-Koch-Institute [81]
