# Supplementary material for: The Role of Transparency, Trust, and Social Influence on Uncertainty Reduction in Times of Pandemics: Empirical Study on the Adoption of COVID-19 Tracing Apps
Source: J Med Internet Res. 2021 Feb 8;23(2):e25893. doi: 10.2196/25893 (PMC7872328; doi:10.2196/25893)
Supplement: Multimedia Appendix 2 [file jmir_v23i2e25893_app2.docx]

Items of the survey and their sources.

| Construct and Source | | Item | Loadings | |
| --- | --- | --- | --- | --- |
| Organizational Transparency Disclosure | | OT_D_1: The information I receive about the Corona App covers completely what I want to know. | .872 | |
|  | (Schnackenberg et al. [63]) |  |  |  |
|  | (α=.874; CR=.877; AVE=.643) | OT_D_2: The information I get about the Corona App covers all topics I want to know something about. | .848 | |
|  |  |  |  |  |
|  |  | OT_D_3: I have all the information I need about the Corona App. | .693 | |
|  |  | OT_D_4: A sufficient amount of information is provided about the Corona App. | .783 | |
| Organizational Transparency Clarity | | OT_C_1: A sufficient amount of information is provided about the Corona App. | .828 | |
|  | (Schnackenberg et al. [63]) | OT_C_2: The information about the Corona App are clear. | .867 | |
|  | (α=.913; CR=.905; AVE=.704) |  |  |  |
|  |  | OT_C_3: The information of the Corona App is comprehensible. | .891 | |
|  |  | OT_C_4: For me, the information about the Corona App are expressed in a linguistically understandable way. | .766 | |
| Organizational Transparency Accuracy | | OT_A_1: The information about the Corona App seem to be true. | .937 | |
|  | (Schnackenberg et al. [63]) | OT_A_2: The information about the Corona App seem correct. | .949 | |
|  | (α=.967; CR=.967; AVE=.880) |  |  |  |
|  |  | OT_A_3: The information about the Corona App seem accurate. | .931 | |
|  |  | OT_A_4: The information about the Corona App seem right. | .936 | |
| Trust of the Government | | TOG_1: I think I can trust state government agencies. | .931 | |
|  | (Bélanger,[53]) |  |  |  |
|  | (α=.921; CR=.921; AVE=.747) | TOG_2: State government agencies can be trusted to carry out online offerings faithfully. | .795 | |
|  |  |  |  |  |
|  |  | TOG_3: I trust state government agencies keep my best interests in mind. | .778 | |
|  |  | TOG_4: In my opinion, state government agencies are trustworthy. | .940 | |
| Social Influence | | SI_1: People who are important to me think that I should use the Corona-Warn-App. | .935 | |
|  | (Venkatesh, et al. [107]) |  |  |  |
|  | (α=.933; CR=.934; AVE=.825) | SI_2: People who influence my behavior think that I should use Corona-Warn-App. | .866 | |
|  |  | SI_3: People whose opinions that I value prefer that I use Corona-Warn-App. | .922 | |
| Initial Trust | | TR_1: The Corona-Warn-App is trustworthy. | .920 | |
|  | (Koufaris & Hampton-Sosa [58]) | TR_2: I trust the Corona-Warn-App keeps my best interests in mind. | .848 | |
|  | (α=.935; CR=.936; AVE=.785) | TR_3: The Corona-Warn-App will keep promises it makes to me. | .879 | |
|  |  | TR_4: I believe in the information that the Corona-Warn-App provides me. | .896 | |
|  |  | TR_5: The Corona-Warn-App wants to be known as one who keeps promises and commitments | dropped | |
| Intention to Use | | IU_1: I intend to use the Corona-Warn-App. | .987 | |
|  | (Venkatesh et al. [52]) | IU_2: I predict I would use the Corona-Warn-App. | .986 | |
|  | (α=.991; CR=.991; AVE=.973) |  |  |  |
|  |  | IU_3: I plan to use the Corona-Warn-App. | .986 | |
| Privacy Risks | |  | t1 | t2 |
|  | (Rauschnabel [44]) | PRPP_1: The Corona-Warn-App would collect too much information about a user. | .736 | .914 |
|  | (α_t1_=.959; CR_t1_=.852; |  |  |  |
|  | AVE_t1_=.535); (α_t2_=.968; CR_t2_=.960; AVE_t2_=.828) | PRPP_2 I would be concerned about my privacy when using the Corona-Warn-App. | .737 | .910 |
|  |  | PRPP_3: I have doubts as to how well my privacy is protected while using the Corona-Warn-App. | .724 | .909 |
|  |  | PRPP_4: My personal information would be misused when the Corona-Warn-App is running. | .735 | .913 |
|  |  | PRPP_5: My personal information would be accessed by unknown parties when using the Corona-Warn-App in my everyday life. | .724 | .905 |
| Social Risks | |  | t1 | t2 |
|  | (Featherman & Pavlou [89]) | SR_1: The thought of not using the Corona-Warn-App causes me concern because some friends would not think well of me. | .803 | .788 |
|  | (α_t1_=.856; CR_t1_=.857; AVE_t1_=.667); (α_t2_=.836; |  |  |  |
|  | CR_t2_=.837; AVE_t2_=.632) | SR_2: Not using the Corona-Warn-App would lead to a social loss for me because my friends and relatives would think less highly of me. | .870 | .829 |
|  |  | SR_3: Not using the Corona-Warn-App will negatively affect the way others think of you. | .774 | .767 |
| Performance Risks | |  | t1 | t2 |
|  | (Featherman & Pavlou [89]) | PR_1: The Corona-Warn-App might not perform well and create problems. | .820 | .869 |
|  | (α_t1_=.914; CR_t1_=.916; |  |  |  |
|  | AVE_t1_=.685); (α_t2_=.933; CR_t2_=.935; AVE_t2_=.742) | PR_2: The security systems built into the Corona-Warn-App are not strong enough to protect my data. | .794 | .820 |
|  |  | PR_3: The likelihood that there will be something wrong with the performance of the Corona-Warn-App and that it will not work properly is high. | .859 | .902 |
|  |  | PR_4: Considering the expected level of service performance of the Corona-Warn-App, it would be a high risk to use the Corona-Warn-App. | .812 | .821 |
|  |  | PR_5: The Corona-Warn-App may not work properly and process the data incorrectly. | .851 | .892 |
| COVID-19 Concerns | |  | t1 | t2 |
|  | (Conway et al. [45]) | CC_1: Thinking about the coronavirus (COVID-19) makes me feel threatened. | .852 | .853 |
|  | (α_t1_=.879; CR_t1_=.884; |  |  |  |
|  | AVE_t1_=.563); (α_t2_=.871; CR_t2_=.876; AVE_t2_=.548) | CC_2: I am afraid of the coronavirus (COVID-19). | .867 | .881 |
|  |  | CC_3: I am not worried about the coronavirus (COVID-19). | .600 | .540 |
|  |  | CC_4: I am worried that I or people I love will get sick from the coronavirus (COVID-19). | .747 | .767 |
|  |  | CC_5: I am stressed around other people because I worry I will catch the coronavirus (COVID-19). | .760 | .710 |
|  |  | CC_6: I have tried hard to avoid other people because I do not want to get sick. | .638 | .632 |
| Actual Use | | Are you using the Corona-Warn-App? |  | |
|  | (Venkatesh et al. [107]) |  |  |  |
